# Supplementary material for: MetaRibo-Seq measures translation in microbiomes
Source: Nat Commun. 2020 Jun 29;11:3268. doi: 10.1038/s41467-020-17081-z (PMC7324362; doi:10.1038/s41467-020-17081-z)
Supplement: Supplementary file 10 — Supplementary Data 7 [file 41467_2020_17081_MOESM10_ESM.zip › File2/Confidence_High_Taxonomy/275495_out.krona.html]

Javascript must be enabled to view this page.

members
magnitude
magnitudeUnassigned
count
unassigned
taxon
rank

275495\_out

84

2
superkingdom
84

1239
phylum
84

class
84
186801

order
84
186802

83
family
186803

genus
83
841

2
species

SRS011084\_contig\_number\_28651SRS024388\_contig\_number\_8373
360807

166486

SRS013158\_contig\_number\_3381SRS013940\_contig\_number\_17309SRS013951\_contig\_number\_16063SRS014287\_contig\_number\_2559SRS014613\_contig\_number\_2407SRS014855\_contig\_number\_17570SRS014923\_contig\_number\_33823SRS014948\_contig\_number\_13128SRS015065\_contig\_number\_31481SRS015264\_contig\_number\_contig-100\_1491.96276SRS015431\_contig\_number\_29757SRS015578\_contig\_number\_4770SRS015579\_contig\_number\_26378SRS015854\_contig\_number\_24631SRS015890\_contig\_number\_19959SRS015960\_contig\_number\_contig-100\_101.140381SRS016267\_contig\_number\_contig-100\_852.66596SRS018623\_contig\_number\_12955SRS018817\_contig\_number\_contig-100\_2048.125790SRS018984\_contig\_number\_10665SRS019068\_contig\_number\_51937SRS019267\_contig\_number\_4996SRS019397\_contig\_number\_9626SRS019685\_contig\_number\_7074SRS021484\_contig\_number\_contig-100\_953.169857SRS024435\_contig\_number\_44231SRS024549\_contig\_number\_contig-100\_1823.43229SRS043001\_contig\_number\_11169SRS043411\_contig\_number\_contig-100\_2719.94060SRS043701\_contig\_number\_17552SRS045739\_contig\_number\_5394SRS048164\_contig\_number\_contig-100\_1588.134740SRS049900\_contig\_number\_contig-100\_1020.114817SRS050422\_contig\_number\_22968SRS050925\_contig\_number\_35623SRS050941\_contig\_number\_12324SRS052027\_contig\_number\_10740SRS054956\_contig\_number\_11233SRS055017\_contig\_number\_33190SRS056273\_contig\_number\_32217SRS056519\_contig\_number\_15808SRS058070\_contig\_number\_30750SRS063040\_contig\_number\_47713SRS063518\_contig\_number\_35331SRS075341\_contig\_number\_24246SRS075716\_contig\_number\_1680SRS076756\_contig\_number\_11225SRS077454\_contig\_number\_18866SRS077552\_contig\_number\_11077SRS077641\_contig\_number\_17456SRS098061\_contig\_number\_13997SRS098514\_contig\_number\_contig-100\_836.4711SRS098644\_contig\_number\_15337SRS098827\_contig\_number\_9878SRS098881\_contig\_number\_25053SRS104084\_contig\_number\_contig-100\_165.79734SRS1041038\_contig\_number\_3701SRS1041130\_contig\_number\_2336SRS104485\_contig\_number\_4860SRS104975\_contig\_number\_10181SRS105153\_contig\_number\_37525SRS1055034\_contig\_number\_11767SRS142503\_contig\_number\_33520SRS142781\_contig\_number\_10489SRS143070\_contig\_number\_contig-100\_34.109855SRS143148\_contig\_number\_20394SRS143417\_contig\_number\_2254SRS143598\_contig\_number\_contig-100\_222.140487SRS143722\_contig\_number\_15968SRS143780\_contig\_number\_31984SRS144362\_contig\_number\_6928SRS144714\_contig\_number\_contig-100\_1379.135756SRS147919\_contig\_number\_7659SRS148091\_contig\_number\_11252SRS148159\_contig\_number\_7964SRS148424\_contig\_number\_26355SRS148721\_contig\_number\_30319SRS149181\_contig\_number\_30108SRS149879\_contig\_number\_3747SRS893341\_contig\_number\_1546SRS893378\_contig\_number\_10223
81
species

541000
1
family


SRS017821\_contig\_number\_20850
1898205
species
1
